# Editorial: CRISPR-Based Genome Editing in Translational Research—2nd Edition

**DOI:** 10.3390/cells15010035

**Published:** 2025-12-24

**Authors:** Jie Xu, Jifeng Zhang, Dongshan Yang

**Affiliations:** Center for Advanced Models for Translational Sciences and Therapeutics, University of Michigan Medical School, 2800 Plymouth Road, Ann Arbor, MI 48109, USA

## 1. Introduction

Genome editing technologies represented by CRISPR/Cas9 (clustered regularly interspaced short palindromic repeats/CRISPR-associated protein 9) have transformed biomedical research and therapeutic development [1,2,3,4,5,6,7,8]. The second edition of the Special Issue “CRISPR-Based Genome Editing in Translational Research” in *Cells*—co-edited by Drs. Jie Xu, Jifeng Zhang, and Dongshan Yang—highlights recent advances in the field, spanning fundamental biological insights to translational applications. We sincerely thank all the authors for their valuable contributions to this Special Issue. The following editorial provides an overview of these contributions and highlights emerging directions in this rapidly evolving field.

## 2. Advances and Applications in CRISPR Genome Editing

### 2.1. Prime Editing: Enhancing Efficacy and Addressing Safety—Where Is the Balance Point?

A featured review by Daliri et al. from the University of Cologne, Germany [9], presents the latest advances in prime editing, with particular emphasis on how this technology modulates cellular DNA repair pathways such as MLH1 (MutL Homolog 1)-mediated mismatch repair (MMR) to optimize efficacy and safety. Unlike conventional CRISPR nucleases, prime editors can introduce diverse single base pair substitutions, small insertions, and deletions without generating toxic DNA double-strand breaks (DSBs) [6,7,8,10]. However, as the authors cautioned, inhibition of the MMR pathway, which normally corrects mismatched nucleotides during DNA replication, may pose potential risks of tumorigenicity. This concern resonates with a recent report showing that inhibition of the non-homologous end joining (NHEJ) pathway, while increasing the frequency of homology-directed repair (HDR) events, also induced substantial genotoxic effects, including large-scale chromosomal rearrangements [11,12].

### 2.2. Disease Modeling with CRISPR: From Zebrafish to Non-Human Primates

CRISPR/Cas9 revolutionized the development of animal models, especially in species where germline transmitting embryonic stem cells are not available, by enabling precise, efficient, and cost-effective genome editing, allowing scientists to replicate human disease mutations with unprecedented capacity [13,14,15,16,17].

This ability is exemplified by the work of Stemerdink et al. from Radboud University, Netherlands, in which they report the successful creation of a zebrafish line harboring a mutation in ADGRV1 in order to model retinal dysfunction in Usher syndrome [18]. This model accelerates both mechanistic research and preclinical therapy development for retinal disorders.

At the other end of the phylogenetic spectrum, CRISPR was applied to engineer rhesus macaque embryos carrying expanded CAG repeats in the huntingtin gene, modeling the genetics of Huntington’s disease [19]. This non-human primate model would bridge the translational gap between rodent studies and human patients, offering an invaluable platform for elucidating disease pathophysiology and accelerating preclinical evaluation of genome editing strategies. Notably, in September 2025, UK researchers reported the first successful treatment of Huntington’s disease using a gene therapy delivered directly to the brain, which reduced disease progression by up to 75% in clinical trials (ClinicalTrials.gov ID NCT04120493). While this achievement offers new hope, its clinical application currently requires an invasive neurosurgical procedure and needs further validation before widespread adoption. The continued development of non-human primate models, as described here, is expected to facilitate therapeutic development for this devastating disorder.

### 2.3. Streamlining Mutant Genotyping with Reduced Costs: A Universal qPCR-Based Platform for Genotyping Gene Editing Animals

One technical challenge associated with CRISPR-mediated gene editing is that it often generates multiple mutation types, making genotyping technically challenging [20,21,22,23]. Fu et al. from the National Institutes of Health in the US, in collaboration with Chinese and Japanese collaborators, developed a simplified genotyping method [24]. This approach, referred to as parallel qPCR-based iGenotype index for mutant genotyping, allows rapid, scalable, and cost-effective identification of animal genotypes after CRISPR or Transcription Activator-like Effector Nuclease (TALEN) genome modifications, without the need for costly DNA sequencing. The authors demonstrated the application in xenopus and zebrafish, but the method should be readily adoptable to other species. The assay promises to dramatically reduce the cost of genotyping edited founder animals, thus expediting the model development process.

## 3. Therapeutic Gene Editing: Emerging Models and Strategies

This Special Issue also includes three comprehensive review articles on how gene editing can contribute towards conquering challenging human diseases, each with a specific focus: cancer, mitochondrial diseases, and Wilson’s Disease [25,26,27].

### 3.1. CRISPR-Assisted Evaluation of Drug Combinations for Treating Cancer

Kim and Lee from St. Jude Research Hospital provides a thorough review on how the CRISPR/Cas system can be applied to screen drug combinations that provide synergistic potential in cancers, especially in neuroblastoma [27]. Drug combinations, referred to as combining two or more FDA-approved drugs, are explored to treat new conditions [28,29,30,31]. The vast number of possible drug combinations, as well as difficulties arising from inflexible dose ratios and incompatible pharmacokinetics in combination therapies, make preclinical evaluations prohibitory. The authors presented CRISPR-based genetic screening as a potential solution, at least partially. By applying CRISPR knockout and activation screens across diverse models, researchers can efficiently prioritize context-specific drug combinations and reveal candidate targets to improve response. It is particularly useful in identifying and confirming the combination of genetic targets. On the other hand, it lacks dosage precision, and therefore may not faithfully reflect the effects of the drugs. Nevertheless, the emerging CRISPR-based approaches hold promise in improving the evaluation of drug combinations towards novel cancer therapies, including those with neuroblastoma.

### 3.2. CRISPR Therapeutics for Monogenic Diseases

The powerful editing capacity makes CRISPR tools the natural choice for correcting pathogenic mutations causing monogenic diseases [32,33,34,35,36,37,38]. Choi et al. from Korea University College of Medicine summarizes the landscape of CRISPR/Cas9 in diagnosis and treatment of Wilson’s disease [25]. Wilson disease is a rare inherited disorder of copper metabolism, primarily caused by mutations in the *ATP7B* gene, which leads to excessive copper accumulation in the liver, nervous system, kidneys, and other organs, resulting in a wide range of potentially irreversible symptoms [39,40,41]. Early and accurate diagnosis remains challenging due to clinical heterogeneity and high genetic mutation variability across populations, making lifelong management with copper-chelating medications and dietary restrictions essential for affected patients.

This review provides a comprehensive overview of the pathophysiology and genetic landscape of Wilson’s disease, detailing both the conventional diagnostic methods and the recent advancements in CRISPR/Cas-based technologies. It examines how CRISPR/Cas systems are being developed for precise, cost-effective, and high-throughput genetic screening, and explores emerging gene therapy strategies utilizing innovative delivery platforms like AAV, lentiviral vectors, and nanoparticles for correcting ATP7B mutations.

Looking forward, the integration of CRISPR/Cas diagnostics and therapeutics holds significant potential to reshape Wilson’s disease management by enabling rapid point-of-care tests and gene editing therapy. However, the authors caution that further research is needed to overcome technical hurdles such as off-target effects, editing efficiency, delivery specificity, and addressing ethical considerations, before these approaches can be confidently translated to clinical practice.

### 3.3. Beyond CRISPR: Mitochondrial Diseases Call for Novel Approaches

CRISPR technology faces significant challenges in treating mitochondrial diseases because mammalian mitochondria lack a reliable mechanism for importing nucleic acids [42,43]. Consequently, guide RNA cannot enter the mitochondrial matrix, making direct mtDNA editing difficult. Hong et al. from Seoul National University, Korea, reviewed current clinical approaches for mitochondrial diseases which include small molecule compounds, conventional gene therapy (not gene editing therapy), and TALEN-mediated genome editing strategies. TALEN, unlike CRISPR/Cas9, is a protein-based gene editing nuclease [26]. While this same feature makes TALEN and another protein-based gene editing nuclease Zinc Finger Nuclease (ZFN) more difficult to design and synthesize and less efficient in creating edits, as compared to Cas9, they remain the only tools currently suitable for mitochondrial genome editing. Importing reliable nucleic acid into mammalian mitochondria, enabling the development of a functional mitochondrial CRISPR/Cas9 system, would substantially advance the field and could be truly transformative.

## 4. Summary and Perspective

CRISPR genome editing has revolutionized translational research by enabling precise modification of DNA across a variety of model organisms and disease contexts. This Special Issue highlights how emerging techniques, such as prime editing and advanced genotyping platforms, are accelerating therapeutic development, enhancing disease modeling, and enabling new approaches for drug evaluation in cancer and rare genetic conditions.

Despite these advances, real-world clinical translation still faces challenges, including off-target effects [44], delivery barriers [45], and ethical considerations [46,47,48]. Continued innovation, especially interdisciplinary efforts to refine genome-editing tools and their applications, will be essential for realizing the full therapeutic potential of CRISPR technology for human health.
